# Unemployment claims in Philadelphia one year after implementation of the sweetened beverage tax

**DOI:** 10.1371/journal.pone.0213218

**Published:** 2019-03-27

**Authors:** Hannah G. Lawman, Sara N. Bleich, Jiali Yan, Michael T. LeVasseur, Nandita Mitra, Christina A. Roberto

**Affiliations:** 1 Philadelphia Department of Public Health, Philadelphia, Pennsylvania, United States of America; 2 Harvard T.H. Chan School of Public Health, Boston, Massachusetts, United States of America; 3 Perelman School of Medicine, University of Pennsylvania, Philadelphia, Pennsylvania, United States of America; Xiamen University, CHINA

## Abstract

**Objectives:**

Possible adverse economic impacts of sweetened drink taxes are a key concern for numerous stakeholders. This study examined changes in unemployment benefit claims filings in Philadelphia compared to its neighboring counties two years prior to and 14 months post implementation of a 1.5 cents per ounce excise tax on sugar- and artificially-sweetened beverages.

**Methods:**

Data were obtained from the Pennsylvania Department of Labor. Interrupted time series analysis was used to determine if there was a change in new monthly unemployment claims filings post-tax implementation in Philadelphia compared to surrounding counties in supermarkets, select potentially affected industries, and in total claims filings across all industries combined.

**Results:**

Results showed there were no statistically significant changes to unemployment claims in Philadelphia compared to neighboring counties for supermarkets (ß = -9.45, 95% CI = -98.11, 79.22), soft drink manufacturers (ß = -0.13, 95% CI = -9.13, 8.88), across other potentially affected industries (ß = 9.16, 95% CI = -488.29, 506.60), or across all industries (ß = -445.85, 95% CI = -4272.39, 3380.68) following implementation of the beverage tax. Unemployment declined similarly in Philadelphia compared to surrounding counties.

**Conclusions:**

Public reports of increased unemployment within the first year following the implementation of the Philadelphia beverage tax are not supported by this analysis. Future work should examine employment outcomes and include longer follow-up periods.

## Introduction

Excise taxes on sweetened drinks are of increasing interest to policymakers for both public health and revenue generation reasons [1,2] and are recommended by the National Academy of Medicine [3] and the World Health Organization [4] to reduce consumption of sugar-sweetened beverages (SSBs). Several cities in the United States and countries across the globe have passed excise taxes on sweetened beverages including Albany, Oakland, San Francisco, and Berkeley, CA, Philadelphia, PA, Boulder, CO, Seattle, WA, Mexico, Saudi Arabia, and Belgium [5,6]. Initial data from Mexico and Berkeley suggest excise taxes may be effective tools for reducing consumption [7] and sales [8–10] of SSBs, and many other localities and countries are considering or pursuing SSB excise taxes [5,6]. However, possible adverse economic impacts of such taxes, including potential job loss, are a key concern for numerous stakeholders. As the popularity of beverage excise taxes as a public health measure grows, research on the wider economic impact of beverage excise taxes has been called for to fully understand their impact [6].

Philadelphia implemented a 1.5 cent per ounce excise tax on distributors of sugar- and artificially-sweetened beverages beginning January 1, 2017. Preliminary evidence from the first two months of the tax suggests that 93% of the tax was passed on to customers at the airport [11] and that odds of daily sugary drink consumption was 40–60% lower but there was no change in continuously measured consumption frequency [12]. Public reports from business owners [13–15] and industry-sponsored reports [16] have included claims about negative economic impacts of the tax. These public reports vary in terms of the reported economic influence, including reports of reduced hours for workers (i.e., reduced employment), voluntary job switching (i.e., change of employment) and involuntarily laying off workers (i.e., increased unemployment) [13–16]. Although the pathway generalizes across industries, using the example of a supermarket there are four conditions that must be met for a beverage tax to generate a detectable effect on unemployment: 1) highly elastic demand for sweetened beverages, 2) low substitution between sweetened beverages and other products sold by a store, 3) profits from sweetened beverages account for a high share of the total profits earned by the store, and 4) total profit loss is so great that the employer has no alternative but to dismiss its employees.

Although reports of negative employment and unemployment impacts in the media are prevalent, there is limited real-world evidence about economic impacts based on implemented beverage tax policies [17], none of which is from the United States. Simulation studies on the cost effectiveness of a beverage excise tax nationally [18] or locally in Philadelphia [19] suggest that beverage excise taxes are cost-saving (i.e., generate more revenue than required to implement the policy), and produce substantial healthcare cost savings, but these studies have not considered broader potential economic impacts, such as increased unemployment. Other available empirical [17] and simulation [20] studies show no changes to employment or unemployment following beverage excise taxes and possible increased employment. Philadelphia is unique among U.S. cities with beverage excise taxes as it has high rates of residents living in poverty compared to other major cities [21] and is the only U.S. city to tax both sugar- and artificially-sweetened drinks. Therefore, the purpose of the current study is to examine changes in unemployment benefit claims filings in Philadelphia compared to its neighboring counties two years prior to and 14 months post-tax implementation.

## Methods

### Data source

Data were obtained from the Unemployment Compensation Program at the Pennsylvania Department of Labor from January 2015 through February 2018 for 11 large counties in Pennsylvania for several industries potentially affected by the beverage tax (see **Table A in** S1 Appendix). There are no ethical or legal restrictions on sharing the de-identified dataset. Data were received fully de-identified. Data represent the number of new claims filed each month per county and per specified North American Industry Classification System (NAICS) code based on the claimant’s county of residency. Our data only reflect new claims and not continuous counts of benefit receipt or denied claims. Unemployment compensation claims (often called unemployment insurance claims) have been used to study numerous economic effects related to unemployment [22,23] supporting its use as a proxy for unemployment. In Pennsylvania, eligibility to file for unemployment compensation includes a) financial eligibility consisting of meeting all three criteria of: earnings of at least $116 per week during at least 18 weeks in the base period, earnings of at least $1,688 during the highest quarter of the base period, and earnings of at least $3,391 total wages during the base period, b) being out of work through no fault of your own, and c) being able, available, and looking for work [24]. A base period is a one-year period including the earliest four of the last five completed quarters of the calendar year. For example, if one applies for unemployment in February 2017, the base period would be October 1, 2015 through September 30, 2016. Unemployment compensation in Pennsylvania is available for up to 26 weeks with a possible extension.

### Procedures

Total claims filings (based on the NAICS 10) along with claims for several selected industries potentially affected by the tax were examined. Individual industries of interest included supermarkets (NAICS 44511) and soft drink manufacturing (NAICS 312111) because these industries have asserted that the tax has led to large job losses [13–15]. Additional food-related industries, which included wholesale groceries, retail groceries, retail vending machines, retail drug stores, retail department stores, restaurants, food service, and bars and taverns (see Table A in S1 Appendix) were summed to create one category of “potentially affected industries.” Data from three counties contiguous to Philadelphia (Delaware, Montgomery, Bucks) were aggregated and used as the comparison group. These counties constitute the Philadelphia suburban area in Pennsylvania and are among the largest and most comparable markets in state outside of Philadelphia. Although the demographic profile of suburban residents is less ethnically/racially diverse and higher-income compared to Philadelphia, the neighboring counties are a conservative control. This is because consumers may try to avoid the tax by shopping in these neighboring areas. This would in turn lead to increased sales and/or decreased unemployment in the neighboring counties, which would result in a larger difference between treatment and control and therefore a greater likelihood to detect rises in unemployment associated with the tax. The other demographically and economically comparable county in Pennsylvania (Allegheny, which contains the city of Pittsburgh) was used in sensitivity analyses as an alternative comparison county.

### Statistical analysis

Descriptive statistics for monthly unemployment claims filings before versus after the tax were calculated. Interrupted time series analysis with the intervention and comparison locations was used to determine if there was a change in monthly unemployment claims filings post-tax implementation using two interactions and all lower order terms and covariates as shown in Eq 1 [25].

$${Unemployment}_{it}=\beta_{0}+\beta_{1}(season)+\beta_{2}(time)+\beta_{3}(Philadelphia)+\beta_{4}(post\;tax)+\beta_{5}(time*Philadelphia)+\beta_{6}(post\;tax*Philadelphia)+e_{it}$$ (1)

Where *i* indicates county and *t* indicates time period; ß_0_ is the intercept interpreted as the expected monthly unemployment claims in neighboring counties prior to the tax; ß_1_ is the set of parameters for 3 dummy coded covariates for season using winter as the reference group; ß_2_ is the parameter for time which was coded as month ranging from 1–38 starting in Jan 2015; ß_3_ is the parameter for binary treatment (Philadelphia or aggregated neighboring counties); ß_4_ represents the parameter for post-tax implementation which was coded as a binary indicator of month before or after tax implementation; ß_5_ is the parameter for the interaction term representing baseline trend differences that is interpreted as the difference in baseline monthly unemployment for Philadelphia before the tax was implemented; ß_6_ is the parameter for the interaction term representing monthly changes in unemployment filings after the tax in Philadelphia compared to neighboring counties and is the primary effect of interest; *e*_*tj*_ is the error term, which may be serially correlated. Correlation over time was accounted for using an AR(1) autocorrelation structure (i.e., *e*_*it*_-1 and *e*_*it*_ are correlated).

Autocorrelation was accounted for using autoregressive process of order one (Durbin-Watson statistics = 1.54, p = .06 for supermarkets, 1.24, p < .01 for soft drink manufacturing, 0.95, p < .01 for potentially affected industries, and 1.55, p < .05 for total). The assumption of parallel trends, which stipulates that the pre-intervention trends in the outcome of interest are comparable for treatment and control locations, was tested using generalized least squares models with the continuous month variable, the county, and the interaction between the two. Separate models for each of the four outcomes were run, and no interactions were significant indicating the parallel trends assumption was met. The current study had 80% power to detect an effect size of Cohen’s d = 0.5 or about a 10% change (approximately 11 average monthly supermarket unemployment claims and 834 average monthly total unemployment claims). Three robustness checks were performed, and results did not change: 1) Allegheny County (which contains Pittsburgh) was used as a comparison, 2) an uncorrelated error structure was specified, and 3) the date of impact on unemployment claims was set to July 2017 instead of Jan 2017 to test whether there were delayed impacts on employment given that some public reports were projecting future job losses at the time of tax implementation. S1 Appendix provides alternative model specifications considered,R code for final models, and results from sensitivity analyses (see Table B in S1 Appendix).

## Results

**Fig 1:**
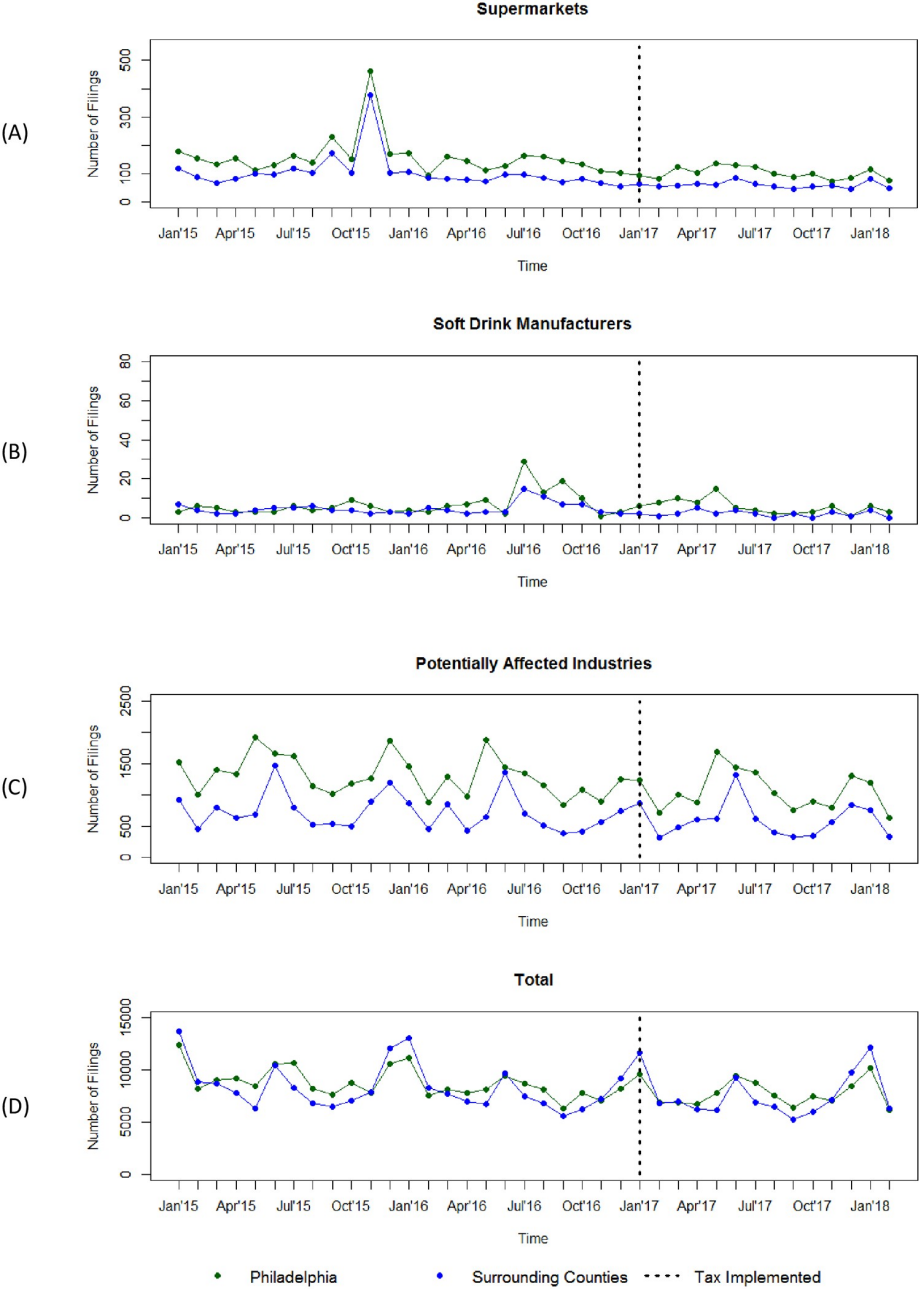
Unadjusted new monthly unemployment benefit claims filings in Philadelphia and surrounding counties, 2015–2018. Supermarkets = NAICS 44551, All Industries = NAICS 10; Potentially Affected Industries = wholesale groceries, soda and ice manufacturing, retail groceries, retail vending machines, retail drug stores, retail department stores, restaurants, food service, and bars and taverns. The Philadelphia beverage tax passed Jun 16, 2016 and was implemented Jan 1, 2017. Two regional supermarket chains closed in 2015 and explain the observed spike in supermarkets panel (A).

Descriptive data for unemployment claims before and after the tax are shown in Table 1 by county. Compared to the two years pre-tax, claims declined similarly in Philadelphia as compared to the surrounding counties for supermarkets, soft drink manufacturing, all potentially affected industries, and total unemployment. Fig 1 shows unadjusted claims by month for Philadelphia and surrounding counties.

**Table 1:** Average new monthly unemployment benefit claims filings in Philadelphia and surrounding counties, 2015–2018.

	Philadelphia	Neighboring Counties	Mean Difference (PHL-NC)
Supermarkets			
Pre-Tax Mean (SD)	158.29 (70.78)	104.38 (62.53)	53.92
Post-Tax Mean (SD)	102.43 (20.71)	60.29 (11.62)	42.14
Mean Difference	-55.86	-44.09	-11.77
Soft Drink Manufacturers			
Pre-Tax Mean (SD)	6.75 (6.19)	4.67 (3.10)	2.08
Post-Tax Mean (SD)	5.64 (3.75)	2.00 (1.57)	3.64
Mean Difference	-1.11	-2.67	1.56
PBT Industries			
Pre-Tax Mean (SD)	1308.46 (317.7)	719.92 (292.56)	588.54
Post-Tax Mean (SD)	1066.93 (310.80)	596.86 (278.40)	470.07
Mean Difference	-241.53	-123.06	-118.47
Total			
Pre-Tax Mean (SD)	8749.17 (1430.64)	8314.58 (2140.27)	434.58
Post-Tax Mean (SD)	7822.86 (1272.99)	7656.43 (2169.06)	166.43
Mean Difference	-926.31	-658.15	-268.15

PHL = Philadelphia; NC = neighboring counties; Supermarkets = NAICS 44551, Soft drink manufacturing = NAICS 312111, All Industries = NAICS 10; Potentially Affected Industries = wholesale groceries, soda and ice manufacturing, retail groceries, retail vending machines, retail drug stores, retail department stores, restaurants, food service, and bars and taverns. The Philadelphia beverage tax was implemented Jan 1, 2017.

Time series analysis results are shown in Table 2. There were no statistically significant changes to unemployment claims in Philadelphia compared to neighboring counties following the implementation of the beverage tax for supermarkets (β = -9.45, SE = 45.24, p = 0.84), soft drink manufacturing (β = -0.13, SE = 4.59, p = 0.98), all potentially affected industries (β = 9.16, SE = 253.80, p = 0.97), or total unemployment claims (β = -445.85, SE = 1952.35, p = 0.82). The large spike in 2015 (prior to tax legislation passage and tax implementation) was due to two regional supermarket chains going out of business (Fig 1A).

**Table 2:** Controlled interrupted time series analysis results of new monthly unemployment benefit claims filing in Philadelphia and surrounding counties, Jan 2015 –Feb 2018.

	Supermarkets		Soft Drink Manufacturers		Potentially Affected Industries		All Industries	
	Est (SE)	p-value	Est (SE)	p-value	Est (SE)	p-value	Est (SE)	p-value
(Intercept)	167.84 (26.90)	<0.01	2.86 (2.73)	0.30	829.11 (150.41)	<0.01	9520.49 (1163.69)	<0.01
Spring	-43.07 (18.51)	0.02	1.2 (1.65)	0.47	-90.49 (101.6)	0.38	815.34 (690.81)	0.24
Summer	-42.63 (18.69)	0.03	0.52 (1.74)	0.77	167.07 (103.55)	0.11	1.60 (730.69)	1.00
Fall	-20.79 (17.79)	0.25	3.87 (1.56)	0.02	-122.53 (97.42)	0.21	-950.83 (650.25)	0.15
Time	-2.95 (1.50)	0.05	0.03 (0.15)	0.83	-7.82 (8.41)	0.36	-89.83 (65.59)	0.18
Philadelphia	55.43 (29.07)	0.06	0.58 (3.13)	0.85	673.7 (164)	0.00	331.45 (1345.40)	0.81
Pre-Post Tax	14.17 (33.79)	0.68	-3.23 (3.45)	0.35	36.97 (189.66)	0.85	874.09 (1464.91)	0.55
Time* PHL	-0.12 (1.99)	0.95	0.1 (0.21)	0.61	-6.8 (11.17)	0.55	7.17 (87.61)	0.94
Pre-Post* PHL	-9.45 (45.24)	0.84	-0.13 (4.59)	0.98	9.16 (253.8)	0.97	-445.85 (1952.35)	0.82

PHL = Philadelphia; TX = Treatment; Supermarkets = NAICS 44551, Soft Drink Manufacturers = NAICS 312111, All Industries = NAICS 10; Potentially Affected Industries = wholesale groceries, soda and ice manufacturing, retail groceries, retail vending machines, retail drug stores, retail department stores, restaurants, food service, and bars and taverns. Spring/Summer/Fall = dummy season covariates; Time = month ranging from 1–38 starting in Jan 2015; PHL/Philadelphia = binary indicator of treatment city; Pre-post Tax = binary indicator of month before or after-tax implementation (Jan 1, 2017); The two interaction terms can be interpreted as baseline trend differences by county and level changes at the time of the tax in Philadelphia, respectively.

## Discussion

As preliminary evidence of the effectiveness of beverage excise taxes in reducing consumption and sales of SSBs is beginning to emerge [7–10], research on the broader economic impacts, including unemployment, of such policies is critical. The aim of the current study was to examine potential changes in new unemployment benefit claim filings in supermarkets, soft drink manufacturers, all industries potentially affected by a beverage excise tax, and total industries in Philadelphia compared to its neighboring counties.

Results showed that filings for new unemployment benefit claims for supermarkets, soft drink manufacturers, all potentially affected industries (e.g., groceries, retail stores, restaurants), and total industries declined similarly in Philadelphia and the surrounding counties following implementation of the beverage tax. By comparison, the closing of a regional supermarket chain in 2015 produced large, visible spikes in unemployment compensation claims. In addition, new unemployment claims filed in soft drink manufacturing, the industry with the potential for a large impact from the tax, remained low throughout the study period (on average 2–7 per month). There were a few months of higher than usual filings in soft drink manufacturing beginning two weeks after passage of the tax. It would be unlikely to observe filings increase due to the tax so close to passage of the law and prior to implementation because this would require employers to have immediately laid off workers and for workers to have immediately started paperwork for unemployment insurance rather than spending some time searching for alternative employment. It is possible that changes in unemployment filings for soft drink manufacturing in Philadelphia relative to neighboring counties were too small to be detected in the current study.

Study results are consistent with previous studies [17,20]. A study conducted two years after Mexico’s 1 peso per liter tax on sugar-sweetened beverages found no changes in national unemployment as a result of the beverage tax and additionally found no employment reductions in commercial food stores or in the manufacturing industry [17]. Results from the current study and previous studies [17,20] differ from industry reports estimating 300 citywide layoffs in supermarkets, 80–100 layoffs in soft drink manufacturing, and an employment decline of 1,192 workers (estimated to be largely in grocery retail) due to the tax [13,15,16]. Although more research on a variety of unemployment and employment indicators is needed, the current study did not find support for the claims of large and precipitate job losses in supermarkets, local soft drink manufacturers, or more broadly following the implementation of the Philadelphia beverage tax.

The Philadelphia beverage tax was implemented during a period when the unemployment rate was falling in Philadelphia and across the country. Results from the current study suggest that, economically, the Philadelphia beverage tax was a relatively minor policy intervention that would be unlikely to invert this trend in the short term, even when considering only local and regional impacts. An alternative interpretation of the current study’s findings may be that employees exiting supermarket, soft drink manufacturing, or other potentially affected industries were absorbed by other sectors and hence unlikely to file for unemployment. Still, there was insufficient evidence of net impacts on unemployment compensation claims following the beverage tax. Although the current study did not have data on employment, results are consistent with findings from a simulation study of the estimated employment impact of sugar-sweetened beverage taxes [20]. This study found that employment growth in government sectors and non-beverage industries offset declines in beverage industry employment such that there may be net employment growth resulting from beverage excise taxes [20]. Studies of the employment impact of tobacco taxes have also concluded that growth in other industries outpaces any employment loss in the tobacco industry and that money previously spent on tobacco tends to be spent on other products [26,27]. The current study expands on previous research of the public health impacts of beverage excise taxes [7–10] by examining one aspect of the broader economic impact of such policies, which is important as their popularity as public health policies is growing [5,6].

Limitations of the current study should be considered. First, data were not available to examine job loss or unemployment that did not result in unemployment claims and thus cannot capture all potential economic impacts related to job loss or job change. Barriers to filing for unemployment compensation, such as navigating the process, may be reasons eligible parties did not file for unemployment claims. However, these factors would not be expected to differ before versus after the tax. Second, it was outside the scope of the current study to examine employment data (unemployment does not account for those who may have withdrawn from the labor market or are employed partially but seeking more work). Still, public reports have equivocated on what specific economic impacts may be associated with tax implementation and have included claims of “job loss” [16] and “layoffs” [14], which may be detected in unemployment compensation claims data. Lastly, data based on the county of employment rather than the county of residence as well as data on other aspects of unemployment, such as the duration of unemployment or claims result (e.g., approved/denied), were not available. However, concerns about error due to reliance on claimant county of residence is somewhat mitigated due to the robustness of sensitivity analyses using Alleghany as an alternative comparison county. Strengths include the use of data going back to 2015, the inclusion of similar comparison counties, the inclusion of several industries potentially affected by a beverage excise tax, and the inclusion of unemployment benefit claims data from the universe of companies and industries in the selected counties. Future work should examine employment outcomes (e.g., reduced employment; part-time seeking full-time employment) and include longer follow-up periods.

In conclusion, public reports of increased unemployment within the first year following the implementation of the Philadelphia Beverage Tax are not supported by this analysis. Although additional economic research is needed, these results suggest that beverage excise taxes may not necessarily produce net changes in involuntary job loss.

## Supporting information

S1 Appendix Model specifications and list of NAICS codes. (DOCX)

S1 Raw Data File (TXT)
