# Supplementary material for: Unemployment claims in Philadelphia one year after implementation of the sweetened beverage tax
Source: PLoS One. 2019 Mar 27;14(3):e0213218. doi: 10.1371/journal.pone.0213218 (PMC6436769; doi:10.1371/journal.pone.0213218)
Supplement: S1 Appendix — (DOCX) [file pone.0213218.s001.docx]

**S1 Appendix. Model specifications and list of NAICS codes.**

*Analytic approach*.

R code for the final analysis models corresponding to the publicly available data can be found below. Alternative model specifications and analytic approaches were considered, and all models produced similar results to the primary analytic approach. First, multilevel models were run using county as the cluster, but due to having only 2 counties this approach was excluded. Second, as suggested by Wagner and colleagues (23), a model was run including a third interaction term between treatment city and the amount of time since treatment was initiated (coded such that months prior to the tax were 0 and post-tax months were coded 1-14). This was to test for a change in the monthly unemployment trends post tax in Philadelphia compared to the aggregated neighboring counties. This approach was excluded out of concern for over-saturating the model and the possibility that this last interaction may absorb much of any true treatment effect. Results are shown in Appendix Table 2 and are consistent with the primary results excluding the third interaction and lower order terms.

*Final R Code*

# Attach package

install.packages(“nlme”) # run this line if you have never used “nlme” package

library(nlme)

# Import data from directory

df <- read.table(paste(directory, "/RawData_Public.txt", sep = ""), header = TRUE, stringsAsFactors = FALSE)

#Data Dictionary:

# Date: month and year

# NAICS: North American Industry Classification System code corresponding to the industry the claims filings are associated with

# NoUnemp: number of new unemployment compensation claims filings in the given month

# County: claimant county of residence (coded as Philadelphia, Bucks, Montgomery, Delaware, Allegheny, and Neighbor [sum of Montgomery, Bucks, Delaware])

# time: months coded 1-38 for Jan 2015 through Feb 2018

# posttax: dummy variable for tax implementation date (1=months from Jan 2017 - Feb 2018; 0=months prior to Jan 2017)

# tx: dummy variable to treatment county (1=Philadelphia; 0=all others)

# txtime: post-tax slop variable coded such that months prior to Jan 1 2017 are 0 and months after are 1, 2, 3…14

# spring: dummy variable for fall season for months Jan-Mar each year

# summer: dummy variable for summer season for months Apr-June each year

# fall: dummy variable for fall season for months July-Sept each year

#Final Models for Lawman et al manuscript "Unemployment claims in Philadelphia one year after implementation of the sweetened beverage tax"

# selecting Philly vs Aggregated Neighboring counties and releveling reference group

df2<- df[df$County=="Philadelphia"| df$County=="Neighbor", ]

df2$County <- as.factor(df2$County)

df2$County <- relevel(df2$County, ref="Neighbor")

#THIS IS THE FINAL MODEL: SUPERMARKETS

analysisdf1 <- df2[df2$NAICS=="44511",]

mfinal1 <- gls(NoUnemp ~ spring + summer + fall + time*County + posttax*County, data = analysisdf1, correlation=corAR1(form = ~ 1 | County)) #generalized least squares model with AR1 correlation structure to account for correlation within county

summary(mfinal1)

confint(mfinal1)

#THIS IS THE FINAL MODEL: SOFT DRINK MANF

analysisdf2 <- df2[df2$NAICS=="312111",]

mfinal2 <- gls(NoUnemp ~ spring + summer + fall + time*County + posttax*County, data = analysisdf2, correlation=corAR1(form = ~ 1 | County)) #generalized least squares model with AR1 correlation structure to account for correlation within county

summary(mfinal2)

confint(mfinal2)

#THIS IS THE FINAL MODEL: ALL PBT INDUSTRIES

analysisdf3 <- df2[df2$NAICS=="PBT Industries",]

mfinal3 <- gls(NoUnemp ~ spring + summer + fall + time*County + posttax*County, data = analysisdf3, correlation=corAR1(form = ~ 1 | County)) #generalized least squares model with AR1 correlation structure to account for correlation within county

summary(mfinal3)

confint(mfinal3)

#THIS IS THE FINAL MODEL: TOTAL

analysisdf4 <- df2[df2$NAICS=="All NAICS",]

mfinal4 <- gls(NoUnemp ~ spring + summer + fall + time*County + posttax*County, data = analysisdf4, correlation=corAR1(form = ~ 1 | County)) #generalized least squares model with AR1 correlation structure to account for correlation within county

summary(mfinal4)

confint(mfinal4)

S1 Appendix Table A. North American Industry Classification System (NAICS) codes for industries potentially affected by a sweetened beverage excise tax.

| 5 Digit NAICS | NAICS Description | Industry Grouping |
| --- | --- | --- |
| 42441 | General Line Grocery Wholesalers | Wholesale Groceries |
| 42442 | Packaged Frozen Food Wholesalers | Wholesale Groceries |
| 42443 | Dairy Products (Exc Dried/Can) Wholesalers | Wholesale Groceries |
| 42444 | Poultry/Poultry Prod Wholesalers | Wholesale Groceries |
| 42445 | Confectionery Wholesalers | Wholesale Groceries |
| 42446 | Fish/Seafood Wholesalers | Wholesale Groceries |
| 42447 | Meat/Meat Prod Wholesalers | Wholesale Groceries |
| 42448 | Fresh Fruit/Vege Wholesalers | Wholesale Groceries |
| 42449 | Other Grocery/Related Prod Wholesalers | Wholesale Groceries |
| 31211 | Soft Drink & Ice Manufacturing | Soda and Ice Manufacture |
| 44511 | Supermarkets (Exc Convenience) Retail | Retail Groceries - Supermarkets |
| 44529 | Other Specialty Food Store Retail | Retail Groceries - Specialty Food Stores |
| 44512 | Convenience Stores Retail | Retail Groceries - Convenience Stores |
| 45421 | Vending Machine Operators Retail | Retail _Vending Machine Companies |
| 44611 | Pharmacies/Drug Stores Retail | Retail - Drug Stores |
| 45211 | Department Stores Retail | Retail - Dept Stores, Superstores |
| 45291 | Warehouse Clubs/Superstore Retail | Retail - Dept Stores, Superstores |
| 72221 | Restaurants Take-Out | Restaurants - Take Out |
| 72211 | Restaurants Full-Service | Restaurants - Full Service |
| 72233 | Mobile Food Services (Trucks) | Food Service - Food Trucks |
| 72231 | Food Serv Concession/Contractor | Food Service - Concessionaires |
| 72232 | Caterers | Food Service - Caterers |
| 72241 | Drinking Places (Alcoholic Bev) | Bars and Taverns |

Note: These industry codes were aggregated to create one measure of potentially affected industries.

S1 Appendix Table B. Controlled interrupted time series analysis results of new monthly unemployment benefit claims filing in Philadelphia and surrounding counties, Jan 2015 – Feb 2018.

|  | Supermarkets | | Soft Drink Manufacturers | | Potentially Affected Industries | | All Industries | |
| --- | --- | --- | --- | --- | --- | --- | --- | --- |
|  | Est (SE) | p-value | Est (SE) | p-value | Est (SE) | p-value | Est (SE) | p-value |
| (Intercept) | 167.11 (28.99) | <0.01 | 2.42 (2.65) | 0.36 | 838.44 (162.67) | <0.01 | 9693.04 (1314.69) | <0.01 |
| Spring | -41.25 (19.29) | 0.04 | 1.20 (1.58) | 0.45 | -86.46 (105.65) | 0.42 | 850.50 (715.14) | 0.24 |
| Summer | -41.35 (19.76) | 0.04 | 0.39 (1.67) | 0.82 | 164.09 (109.19) | 0.14 | 87.63 (763.43) | 0.91 |
| Fall | -19.28 (18.52) | 0.30 | 3.73 (1.50) | 0.02 | -122.86 (101.29) | 0.23 | -926.4 (672.16) | 0.17 |
| Time | -2.99 (1.69) | 0.08 | 0.07 (0.16) | 0.64 | -8.59 (9.56) | 0.37 | -106.55 (80.27) | 0.19 |
| Philadelphia | 52.94 (32.73) | 0.11 | -0.89 (3.12) | 0.78 | 671.94 (185.41) | 0.00 | 268.50 (1594.66) | 0.87 |
| Pre-Post Tax | 11.91 (39.85) | 0.77 | -2.71 (3.53) | 0.44 | 24.71 (222.54) | 0.91 | 762.92 (1677.01) | 0.65 |
| Time Since TX | 0.42 (4.01) | 0.92 | -0.18 (0.38) | 0.64 | 3.54 (22.62) | 0.88 | 56.58 (189.14) | 0.77 |
| Time* PHL | 0.08 (2.29) | 0.97 | 0.23 (0.22) | 0.29 | -6.64 (12.94) | 0.61 | 12.16 (109.23) | 0.91 |
| Pre-Post* PHL | -4.31 (53.35) | 0.94 | 2.17 (4.73) | 0.65 | 7.93 (298.08) | 0.98 | -476.84 (2245.74) | 0.83 |
| Time Since TX*PHL | -1.21 (5.63) | 0.83 | -0.64 (0.53) | 0.23 | -0.35 (31.79) | 0.99 | -9.80 (266.46) | 0.97 |

PHL=Philadelphia; TX=Treatment; Supermarkets = NAICS 44551, Soft Drink Manufacturers = NAICS 312111, All Industries = NAICS 10; Potentially Affected Industries = wholesale groceries, soda and ice manufacturing, retail groceries, retail vending machines, retail drug stores, retail department stores, restaurants, food service, and bars and taverns. Spring/Summer/Fall = dummy season covariates; Time = month ranging from 1-38 starting in Jan 2015; PHL/Philadelphia = binary indicator of treatment city; Pre-post Tax = binary indicator of month before or after-tax implementation (Jan 1, 2017); Time since treatment = months prior to tax coded 0 and post-tax months coded 1-14. The three interaction terms can be interpreted as baseline trend differences by county, level changes at the time of the tax by county, and trend changes after the tax by county, respectively.
